# Single-cell RNA sequencing: new insights for pulmonary endothelial cells

**DOI:** 10.3389/fcell.2025.1576067

**Published:** 2025-06-17

**Authors:** Ying Yang, Mengyuan Wang, Xiao Qiu, Rui Yang, Chengfang Yao

**Affiliations:** ^1^ Shandong University of Traditional Chinese Medicine, Jinan, China; ^2^ The Central Hospital Affiliated to Shandong First Medical University, Jinan, China; ^3^ School of Clinical and Basic Medicine, Shandong First Medical University (Shandong Academy of Medical Sciences), Jinan, China

**Keywords:** single-cell RNA sequencing, pulmonary endothelial cells, biomarkers, subcluster characterization, lung pathology

## Abstract

Pulmonary endothelial cells (PECs) are indispensable for sustaining lung microenvironmental homeostasis and exert significant influence across a spectrum of pulmonary pathologies. Single-cell RNA sequencing (scRNA-seq) has fundamentally transformed conventional paradigms surrounding PECs, unveiling novel perspectives on their roles in both physiological and pathological lung conditions. This technology provides critical insights into the phenotypic diversity and distinct molecular signatures of PECs, underscoring their substantial heterogeneity in structure, function and gene expression, which is contingent upon their spatial localization within the lung microenvironment. The advancements in scRNA-seq have catalyzed remarkable progress in the therapeutic management of pulmonary pathophysiology, facilitating breakthroughs in the identification of cellular subpopulations, functional characterization and discovery of innovative therapeutic targets. In this review, we systematically synthesize the markers and subclusters of PECs as delineated by scRNA-seq, elucidate their applications in normal and pathological lung contexts, and propose future directions regarding molecular mechanisms and therapeutic interventions targeting PECs.

## 1 Introduction

Pulmonary endothelial cells (PECs) are pivotal in maintaining physiological lung function and participating in the pathogenesis of various pulmonary diseases (Jambusaria et al., 2020; Goldenberg and Kuebler, 2015). Pulmonary endothelial dysfunction, manifested through multiple interconnected pathological alterations including barrier hyperpermeability, sustained inflammatory activation, ROS-mediated apoptosis, endothelial-mesenchymal transition (EndMT), and metabolic reprogramming constitutes a hallmark of progressive vascular remodeling in both acute and chronic respiratory disorders (Patterson and Lum, 2001; Li et al., 2017; Redetzky et al., 2014). These maladaptive changes synergistically disrupt pulmonary vascular homeostasis, driving disease progression in clinical entities such as coronavirus disease 2019 (COVID-19) (Yamaoka-Tojo, 2020a; Yamaoka-Tojo, 2020b; Bonaventura et al., 2021), lung malignancies (Patterson and Lum, 2001), acute lung injury (Xu and Zhou, 2020), chronic obstructive pulmonary disease (COPD) (Takahashi et al., 2013; Green, 2020; Green and Turner, 2017), pulmonary arterial hypertension (PAH) (Goyanes et al., 2020; Rounds and Harrington, 2019; Hudson and Farkas, 2021), and idiopathic pulmonary fibrosis (IPF) (Ryter and Rosas, 2022).

PECs constitute a highly heterogeneous and functionally versatile population residing along the luminal surfaces of pulmonary vasculature and lymphatics. While they retain canonical endothelial functions such as maintenance of vascular homeostasis, PECs exhibit lung-specific phenotypic and functional specializations, which include facilitating efficient gas exchange, preserving alveolar-capillary barrier integrity, modulating angiogenic processes, orchestrating immuno-inflammatory responses, and mediating tissue repair following pulmonary injury and infection (Maruhashi et al., 2019; Hennigs et al., 2019; Niethamer et al., 2020; Huertas et al., 2018). Notably, PECs function as an active metabolic and endocrine interface, capable of synthesizing, metabolizing, and secreting a wide repertoire of bioactive molecules such as nitric oxide (NO), prostaglandin E2 (PGE2), arachidonic acid derivatives, von Willebrand factor (vWF), endothelin-1 (ET-1), and intercellular adhesion molecule-1 (ICAM-1), which exert autocrine and paracrine regulatory effects on neighboring cells and systemic physiology (Goldenberg and Kuebler, 2015; Emin et al., 2012). Moreover, PECs express an extensive array of surface receptors, including cytokines, chemokines and growth factors, thereby enabling precise sensing and integration of environmental cues. Insights from single-cell transcriptomic atlases of the human lung reveal that PECs are enriched in signaling molecules such as C-X-C Motif Chemokine Ligand 12 (CXCL12), Ephrin-B2 (EFNB2), Semaphorin-3G (SEMA3G), and Vascular Endothelial Growth Factor A (VEGFA), alongside key components of the nitric oxide signaling axis, including Nitric Oxide Synthase 1 (NOS1), Phosphodiesterase 3A (PDE3A), and Phosphodiesterase 4D (PDE4D), which are critically involved in the fine-tuning of pulmonary vascular tone (Schupp et al., 2021).

PECs play a critical role in mediating the pulmonary immune response (Abedi et al., 2020; Metkus et al., 2022). Under inflammatory stimuli, PECs undergo either Type I or Type II activation, mediated by G-protein-coupled receptors (GPCRs) and pro-inflammatory cytokines such as tumor necrosis factor (TNF) and interleukin-1 (IL-1), respectively (Strassheim et al., 2020). Activated PECs secrete IL-6 and transforming growth factor-beta (TGF-β), which differentially regulate T helper cell populations: IL-6 promotes Th17 cell activation while TGF-β suppresses Th1 cell differentiation (Gao et al., 2020). These activated PECs facilitate leukocyte recruitment and migration, enabling immune cell infiltration into inflammatory foci within the lungs to combat pathogens, neoplasms, or infectious agents (Middleton et al., 2002). The single-cell transcriptome atlas of murine endothelial cells (ECs) has revealed a significant enrichment of immunoregulatory signatures in PECs, notably the overexpression of major histocompatibility complex (MHC) class II genes, suggesting their potential involvement in immune surveillance (Kalucka et al., 2020).

Single-cell RNA sequencing (scRNA-seq) enables transcriptomic profiling at single-cell resolution, allowing for precise quantification of mRNA transcripts, detailed characterization of cellular states and regulatory networks, elucidation of developmental trajectories, and comprehensive deconvolution of cellular heterogeneity within mixed populations (Zieg et al., 2017; Vieira et al., 2019; Shi and Cao, 2023). PECs exhibit striking heterogeneity across morphological architecture, functional profiles and transcriptomic signatures, with these variations being intimately governed by their spatiotemporal anatomical niches and dynamic microenvironmental cues. Elucidating these distinct endothelial identities is essential, as they execute specialized biological functions and contribute decisively to the initiation, progression, and resolution of a broad spectrum of pulmonary pathologies (Gomez-Salinero and Rafii, 2018; Caramelo et al., 2023). Investigating the phenotypic diversity and functional alterations of individual PECs-each possessing unique biological characteristics is imperative for understanding their roles in lung disease progression (Gomez-Salinero and Rafii, 2018; Caramelo et al., 2023). While conventional techniques such as microscopy and flow cytometry enable PEC classification, they fail to provide a comprehensive mechanistic understanding of PEC function (Nishino et al., 2021; Bacon et al., 2023; Neubauer and Zieger, 2022). Through integration with advanced computational and bioinformatic frameworks, scRNA-seq facilitates the identification of novel PECs subpopulations and the dissection of their distinct molecular and functional attributes.

This review offers a comprehensive synthesis of the current understanding of PECs, encompassing their biological characteristics, molecular markers, and subcluster classifications. We further highlight the application of scRNA-seq in elucidating endothelial heterogeneity within the pulmonary microenvironment, with a particular focus on its relevance to both physiological and pathological conditions. Additionally, we explore the translational implications of scRNA-seq-based discoveries for the diagnosis, management, and therapeutic targeting of lung diseases.

## 2 Markers of PECs in scRNA-seq

### 2.1 Marker selection for PEC identification

In scRNA-seq studies, precise marker selection is essential to accurately isolate ECs from lung tissue. Typically, multiple endothelial markers are employed simultaneously to define PECs. This article reviews the application of PEC-specific markers across various scRNA-seq studies (Table 1). Schupp et al. compiled six distinct scRNA-seq datasets, identifying 12,563 ECs within an integrated lung dataset based on canonical endothelial markers: Platelet Endothelial Cell Adhesion Molecule 1 (PECAM1)/Cluster of Differentiation 31 (CD31), Cadherin 5 (CDH5)/Vascular Endothelial Cadherin (VE-cadherin), Claudin 5 (CLDN5), and Erythroblast Transformation Specific Related Gene (ERG) (Schupp et al., 2021). In the context of lung adenocarcinoma scRNA-seq analyses, Kim et al. annotated canonical markers such as PECAM1, CLDN5, Fms-like Tyrosine Kinase 1 (FLT1), and Receptor Activity Modifying Protein 2 (RAMP2) to delineate PEC lineages (Kim et al., 2020). In human lung scRNA-seq studies, researchers sorted PECs based on CD31^+^ expression, epithelial cells via Epithelial Cell Adhesion Molecule positive (EPCAM^+^), immune cells through CD45^+^, and stromal populations using EPCAM^−^CD31^−^CD45^−^ criteria (Travaglini et al., 2020). Several investigations focused on defining PECs by integrating positive and negative marker expressions. Goveia et al. sequenced 56,771 ECs from human/mouse (peri)-tumoral lung tissues and cultured human lung tumor endothelial cells (TECs) using CD45^−^and CD31^+^ markers (Goveia et al., 2020). Research suggests that selecting ECs based on CD45^−^and ICAM2^+^ or CD31^+^ criteria yields more robust results compared to the conventional sole reliance on CD31 (Vila et al., 2020). Kalucka et al. employed PECAM1 and CDH5 as endothelial markers while excluding smooth muscle cell marker—Actin Alpha 2 (Acta2), fibroblast marker—Collagen Type I Alpha 1 Chain (Col1a1), erythrocyte markers—Hemoglobin Subunit Alpha 1 (Hba-a1), Hba-a2, and Hemoglobin Subunit Beta (Hbb-bs), pericyte marker—Platelet Derived Growth Factor Receptor Beta (Pdgfrb), or immune cell marker—Protein Tyrosine Phosphatase Receptor Type C (Ptprc) (Kalucka et al., 2020). In certain studies, lung-specific markers were identified using Green Fluorescent Protein (GFP) in transgenic reporter mice. For instance, Cldn5-Bacterial Artificial Chromosome (BAC)-GFP was utilized to label ECs in mouse brain and lung tissues, while Pdgfrb-BAC-eGFP and Chondroitin Sulfate Proteoglycan 4 (Cspg4)-DsRed labeled mural cells, and Pdgfra-H2B-GFP marked fibroblasts. A total of 147 pan-endothelial marker genes were identified in Cldn5-BAC-GFP-labeled ECs, demonstrating the efficacy of this approach in accurately capturing endothelial populations (He et al., 2018). Jambusaria et al. employed an endothelial-specific RiboTag transgenic mouse model to isolate ECs and investigate their heterogeneity across diverse tissue contexts (Jambusaria et al., 2020).

**TABLE 1:** Cell surface markes and subclusters in PECs.

Species	Vivo/vitro	Cells origin	Models	Cells number	Cell surface marker	Subclusters	Ref.
human	vivo	Lung tumor ECs/Peritumor Lung ECs	healthy/tumorous	12,323 hTEC 8,929 hPNEC	CD45 negative, CD31 positive	13 subclusters: arteries, postcapillary, alveolar typeI, Alveolar typell, scavenging,activated, intermediate,tip cell, immature, activated Pcv,lymphatics hpNEC lymphatics hTEC,patient #5 specific	Goveia et al. (2020)
murine	vivo	lung ECs	normal/tumorous	29,007 mNECs and mTECs	CD45 negative, CD31 positive	16 subclusters: arteries, veins, capillaries typeI, capillaries typell, lymphatics,TEC capillaries, proliferating,tip cell, immature,large veins, postcap.veins, neophalanx, activated arteries, interferon, breach cell,pre-breach cell	
human	vitro	cuured lung ECs	lung tumor ECs	6,512	CD45 negative, CD31 positive	4 subclusters: Tip EC, proliferating,endothelial-to-mesenchymal transition, intermediate	
human	vivo	lung ECs	datasets	12 563	PECAM1 (CD31), CDH5 (VE-cadherin), CLDN5, ERG	6 subclusters: lymphatic ECs, arterial, capillary (avWFneg/EMCNhigh/EDNRBpos population, avWFpos/EMCNlow/EDN1pos population), venous (COL15A1pos and COL15A1neg) aerocyte	Schupp et al. (2021)
mice	vivo	mice lung vascular cells	healthy	1,504	Cldn5(BAC)-GFP	8 lung ECs subclusters: aEC,cEC1,cEC,cEC2,capilEC,EC1, EC2,LEC	Vila et al. (2020)
mice	vivo	mice lung ECs	healthy and cre recombination	4,278	CD45 negative, CD31 and ICAM2 positive	4 subclusters: Car4 EC, Plvap EC, Vwf EC (Gja5 or Nr2f2), and Prox1 EC	Vila et al. (2020)
human	vivo	lung tumor ECs	healthy/lung adenocarcinoma	2,107	PECAM1,CLDN5,FLT1,RAMP2	5 subclusters: Tumor ECs,Tip-like ECs, Stalk-like ECs, Lymphatic ECs,EPCs	Kim et al. (2020)
mice	vivo	mice lung ECs	healthy	13,862	Pecam1, Kdr, Cdh5, and Tek (Tie2)	not mentioned	Goveia et al. (2020)
mice	vivo	mice lung ECs	healthy	not mentioned	Pecam1, Cdh5 and excluded Acta2, Col1a1, Hba-a1, Hba-a2, Hbb-bs	5 subclusters: artery, capilary 1, capillary 2, Vein, lymphatic	Kalucka et al. (2020)
mice	vivo	mice lung ECs	RiboTag EC (Cdh5CreERT2/^+^; Rpl22HA/^+^) transgenic mice	1,071	Slc6a1, Slco2a1, Emp2, Atp2a3, Epcam Itgal, Il3ra, St14	not mentioned	Gomez-Salinero and Rafii (2018)
			databases	not mentioned	CD31,CDH5		
human	vivo	hman lung ECs	lung cancer	8,223	not mentioned	6 subclusters: tip cells, high endothelial venules (HEVs),venous ECs,capillary, arterial, lymphatic ECs.	Gillich et al. (2020)
human	vivo	human lung ECs	lung adenocarcinoma	3,381	RAMP2	6 subclusters:extra-alveolar capillary ECs(Endo-C1; Endo-C5),alveolar cECs(Endo-C2),tumor ECs (Endo-C3)arterial ECs (Endo-C4),lymphatic ECs (Endo-C6)	Petegrosso and Kuang (2020)
human	vivo	human lung cells	healthy development	75,000	VWA1,HSPG2	9 subclusters: artery, vein,capillary aerocyte, general capillary cell, capillary intermediate 1 cell, capillary intermediate 2 cell, bronchial vessel 1 cell, bronchial vessel 2 cell, bronchial vessel 1 cell	Jiang et al. (2016)
rat	vivo	lung ECs	healthy/BLM treated	2,181	CD31, CD34, CD144 Vegfr, Vwf, and Pecam1	5 subclusters: cluster a, b, c,d,e	Terpos et al. (2020)

### 2.2 Marker utilization for PEC subcluster classification

In scRNA-seq analyses, PECs within distinct subtypes—such as arterial, venous, capillary, and lymphatic ECs—exhibit unique marker profiles for identification. We have compiled a summary of markers corresponding to various PEC subtypes in Table 2. Vila et al. utilized Gap Junction Alpha-5 Protein (Gja5), Nuclear Factor Erythroid 2-Related Factor 2 (Nrf2), and Prospero Homeobox Protein 1 (Prox1) as specific markers for arterial, venous, and lymphatic ECs, respectively (Vila et al., 2020). Goveia et al. employed multiple markers to validate endothelial classification in non-small cell lung cancer, using CD31^+^/C-X-C Motif Chemokine Receptor 4 (CXCR4), vWF, and EFNB2 to differentiate high-tip cells, veins, and arteries in human TECs. Additionally, Selectin P (SELP) and C-C Motif Chemokine Ligand 14 (CCL14) were identified as signatures for activated postcapillary vein ECs and activated postcapillary vein hTECs, respectively (Goveia et al., 2020).

**TABLE 2:** The markers of different subclusters of PECs.

Species	Vivo/vitro	Arterial	Venous	Capillary	Lymphatic	Postcapillary vein ECs	Extra-alveolar capillary ECs	Tip cells	High endothelial venules (HEVs)	Tumor ECs	Ref
human	vivo	GJA5	Nr2f2	not mentioned	PROX1	not mentioned	not mentioned	not mentioned	not mentioned	not mentioned	Vila et al. (2020)
human	vivo	EFNB2	VWF	not mentioned	not mentioned	SELP	not mentioned	CD31+/CXCR4	not mentioned	CCL14	Goveia et al. (2020)
human	vivo	FBLN5, GJA5	SELP	CA4, CD36	PROX1, PDPN	not mentioned	not mentioned	ESM1, NID2	ACKR1	PLVAP IGFBP7	Gillich et al. (2020)
human	vivo	GJA5, FBLN5	SELP	not mentioned	PDPN CCL21	not mentioned	EDN1 SLC6A4, EDN1,CCL2	ESM1,NID2	ACKR1	IGFBP7, PLVAP	Petegrosso and Kuang (2020)
human	vivo	EFNB2, SOX17, BMX, SEMA3, HEY1, LTBP4 FBLN5, GJA5, GJA4	NR2F2, VCAM1 ACKR1, SELP	CA4,PRX, RGCC, SPARC, SGK1, TMEM100	PROX1, LYVE1, FLT4, PDPN	not mentioned	not mentioned	not mentioned	not mentioned	not mentioned	Schupp et al. (2021)
mice	vivo	GJA5,GJA4, CXCL12	COL15A1 VWA1, SELP, Ackr1, NR2F2	Car4,PRX, SGK1	not mentioned	not mentioned	not mentioned	not mentioned	not mentioned	not mentioned	Schupp et al. (2021)

### 2.3 Top and novel PEC markers identified via scRNA-seq

ScRNA-seq analyses have revealed top marker genes expressed in distinct tissue contexts, such as Peptidoglycan Recognition Protein 1 (Pglyrp1), Lipocalin 2 (Lcn2), and Transmembrane Protein 100 (Tmem100), which are uniquely enriched in specific tissues like the brain, testis, and lung (Kalucka et al., 2020). Jambusaria et al. identified the top 10 surface markers of PECs, including Solute Carrier Family 6 Member 1 (Slc6a1), Solute Carrier Organic Anion Transporter Family Member 2A1 (Muc1), Lymphotoxin Beta (Ltb), Solute Carrier Organic Anion Transporter 2A1 (Slco2a1), Epithelial Membrane Protein 2 (Emp2), ATPase Sarcoplasmic/Endoplasmic Reticulum Ca^2+^ Transporting 3 (Atp2a3), Epcam, Integrin Subunit Alpha L (Itgal), Interleukin 3 Receptor Subunit Alpha (IL3ra), and Suppression of Tumorigenicity 14 (St14) (Jambusaria et al., 2020). Paik et al. investigated the transcriptional landscapes of ECs across organs via scRNA-seq, identifying top 10 genes expressed in PECs, such as Glycosyltransferase-Like Protein 1 (Grtp1), Adrenoceptor Beta 1 (Adrb1), Sodium Voltage-Gated Channel Alpha Subunit 7 (Scn7a), Tmem100, 15-Hydroxyprostaglandin Dehydrogenase (Hpgd), Forkhead Box F1a (Foxf1a), Non-Catalytic Region of Tyrosine Kinase Adaptor Protein Associated Protein 5 (Nckap5), Ras Guanine Nucleotide Exchange Factor 1A (Rasgef1a), Foxf1 Adjacent Noncoding Developmental Regulatory RNA (Fendrr), and Periaxin (Prx) (Paik et al., 2020). Jambusaria et al. further utilized the RiboTag EC (Cdh5CreERT2^+^, Rpl22HA^+^) transgenic model to identify the top 10 most abundant genes in PECs, including AHNAK Nucleoprotein (Ahnak), Microtubule-Actin Crosslinking Factor 1 (Macf1), Actin Beta (Actb), Surfactant Protein C (Sftpc), Spectrin Beta Non-Erythrocytic 1 (Sptbn1), Hypoxia Inducible Factor 2 Alpha (Hif2a), Stearoyl-CoA Desaturase 1 (Scd1), Filamin A (Flna), Adhesion G Protein-Coupled Receptor F5 (Adgrf5), and Low-Density Lipoprotein Receptor Related Protein 1 (Lrp1) (Jambusaria et al., 2020). Additionally, scRNA-seq has uncovered novel pulmonary endothelial markers. Sabbagh et al. identified two new PEC markers, Sodium Voltage-Gated Channel Alpha Subunit 7 (Scn7a) and Sodium Voltage-Gated Channel Beta Subunit 3 (Scn3b), which showed comparable enrichment to angiotensin-converting enzyme (ACE) in PECs, suggesting their potential regulatory roles in ACE function (Sabbagh et al., 2018).

## 3 Subclusters of PECs in scRNA-seq

### 3.1 Anatomical location-based classification of PEC subclusters

ECs exhibit inherent heterogeneity, influenced by factors such as tissue-specific biochemical signals, transcriptional programs, mechanical forces, and metabolic processes (Butler and Bhatnagar, 2019; Potente and Makinen, 2017). Similarly, PECs demonstrate distinct heterogeneities based on their anatomical locations. For instance, PECs are categorized into macrovascular endothelium (maEC) and microvascular endothelium (miEC). maECs reside in large arteries and veins responsible for blood transport to and from the lungs, while miECs are located in capillaries surrounding alveoli where gas exchange occurs. These subtypes differ in morphology, permeability, and responsiveness to stimuli (Kostyunina et al., 2023). For example, maECs exhibit enhanced resistance to shear stress and hypoxia, whereas miECs play a more pronounced role in inflammation and angiogenesis (Niethamer et al., 2020; Plebani et al., 2023; Huertas et al., 2013).

### 3.2 Subcluster classification of PECs via scRNA-seq

Beyond broad categories such as lymphatic, capillary, arterial, and venous ECs, scRNA-seq provides a granular classification of PECs and deeper insights into their heterogeneity. We have compiled the subclusters of PECs in Table 1. He et al. described a scRNA-seq dataset delineating vascular and vessel-associated cell subtypes in mouse brain and lung tissues, featuring 1,504 single-cell transcriptomes from mouse lungs. The analysis identified 17 clusters, including eight PEC subclusters: aEC, cEC1, cEC, cEC2, capilEC, EC1, EC2, and LEC, which correspond to arterial, venous, capillary, and lymphatic ECs (He et al., 2018). A recent study reported that PECs in human lung adenocarcinomas consist of multiple subtypes: extra-alveolar capillary ECs divided into two subclusters based on SLC6A4^+^ and C-C Motif Chemokine Ligand 2 positive (CCL2^+^) expression, and tumor ECs also divided into two subclusters via SLC6A4^+^ and CCL2^+^ expression, including tip ECs and high endothelial venules (HEVs). Furthermore, analysis of highly expressed genes and enriched pathways in each subcluster revealed associations with lymphocyte homing, angiogenesis, and extracellular remodeling (Xing et al., 2021). ScRNA-seq analyses of peritumoral lung tissues in humans and mice, alongside cultured human lung TECs, have explored phenotypic variations across species and models. This study identified 13 subclusters in human PECs, 16 subclusters in mouse PECs, and 4 subclusters in cultured lung tumor ECs. Notably, only tip tumor endothelial cells (tip TECs) exhibited conserved markers consistent across both species and models (Goveia et al., 2020). Several studies have not only delineated PEC subclusters but also characterized their functions based on gene expression profiles within each subpopulation. Specifically, in the lung, veins upregulate distinct metabolic markers, differentiating them from other vascular beds (Kalucka et al., 2020). The heterogeneity observed in control and bleomycin-induced rat lungs was categorized into five clusters: Cluster I exhibited high Neurotrophic Tyrosine Kinase Receptor 2 (Ntkr2) and CXCL12 expression with low Nitric Oxide Synthase 3 (NOS3), Caveolin 1 (CAV1), Matrix Gla Protein (MGP), and SELP levels; Cluster II showed no significant marker gene expression differences; Cluster III displayed notable Atypical Chemokine Receptor 1 (Ackr1), Apolipoprotein L Domain Containing 1 (Apold1), and Vascular Cell Adhesion Molecule 1 (VACM-1) expression expression; Cluster IV was characterized by elevated Nitric Oxide Synthase 3 (Nos3), Caveolin 1 (Cav1), and Matrix Gla Protein (Mgp) with low Ntkr2 and CXCL12 levels; and Cluster V demonstrated high Interleukin 33 (IL33) and SELP expression (Li et al., 2021).

### 3.3 Novel PEC subclusters expressing specific genes

Certain PEC subclusters are distinguished by the expression of specific genes. Schupp et al. confirmed six human PEC subclusters: pulmonary-venous ECs exhibited Collagen Type XV Alpha 1 Chain negative (COL15A1^−^) in lung parenchyma and systemic-venous ECs (COL15A1^+^) in airways and visceral pleura; two capillary EC populations, aerocytes defined by Endothelin Receptor Type B (EDNRB), Sclerostin Domain Containing 1 (SOSTDC1), and T-box Transcription Factor 2 (TBX2) expression, and general capillary ECs (Schupp et al., 2021). Combined scRNA-seq and histological staining revealed that lung capillaries consist of two distinct populations: VWFneg/EMCNhigh/EDNRB1pos and VWFpos/EMCNlow/EDN1pos (Schupp et al., 2021). Niethamer et al. identified a newly described population termed carbonic anhydrase 4 (Car4)-high ECs, which exhibit elevated Car4 expression—a key enzyme catalyzing the reversible hydration of CO_2_. These Car4-high ECs cluster with upregulated expressions of Car4, EDNRB, Kdr, and CD34 but comparable levels of other EC markers such as PECAM1, Plvap, Gpihbp1, and Vwf. Car4-high ECs possess a distinct gene signature, with ligand-receptor analysis suggesting their readiness to receive reparative signals from alveolar type I cells. Notably, these cells are predominantly found in regenerating alveolar regions following acute lung injury (Niethamer et al., 2020; Westphalen et al., 2012). Recent reports also indicate that Car4 ECs specifically express Apln, alongside tip EC genes such as Plaur, Serpine1, Sirpa, Piezo2, and Chst1 identified via scRNA-seq. Conversely, Plvap ECs specifically express stalk cell markers like Aplnr and Tek (Tie2). However, the correlation between Car4/Plvap and tip/stalk ECs does not extend to other established markers such as Esm1/Dll4 for tip ECs or Hes1/Flt1 for stalk ECs (Vila et al., 2020). Gillich et al. systematically characterized adult mouse lung PEC diversity via scRNA-seq, concluding that the alveolar capillary network comprises two intermingled yet stable cell types: gCap (general capillary cells) and aCap (aerocytes) (Gillich et al., 2020).

## 4 Applications of scRNA-seq in normal and diseased lungs

**FIGURE 1:**
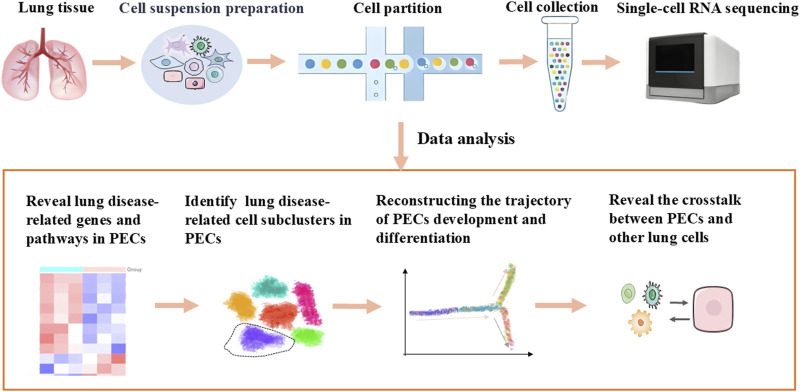
Application of scRNA-seq of PECs in normal and diseased lungs.

ScRNA-seq is capable of revealing lung disease-related genes and pathways, identifying PEC subclusters linked to pathology, elucidating crosstalk between PECs and other lung cells under pathological conditions, delineating spatial distribution and developmental trajectories of PECs, and detecting rare or transient cell states obscured in bulk analyses, which summaried in Figure 1 (Travaglini et al., 2020; Petegrosso and Kuang, 2020; Jiang et al., 2016).

### 4.1 Identification of disease-associated genes and pathways in PECs

ScRNA-seq studies have demonstrated that distinct EC genotypes exhibit unique transcriptional profiles and metabolic pathways under health and disease conditions (Qian et al., 2020), identifying aberrant gene expression patterns and specific subclusters in lung diseases, offering valuable references for discovering potential therapeutic targets (Chen et al., 2022). Novel EC surface markers present opportunities for tissue-specific drug delivery (Paik et al., 2020).

Researchers from Belgium employed scRNA-seq to conduct a comprehensive analysis of thousands of ECs in preclinical models of age-related lung cancer and macular degeneration. Their findings revealed consistent upregulation of genes and metabolic pathways during angiogenesis in diseased lungs. Furthermore, they concluded that targeting EC metabolism represents a promising strategy for preventing pathological blood vessel formation, such as in lung cancer (Rohlenova et al., 2020). ScRNA-seq analysis of mouse PECs in lung cancer uncovered previously unknown metabolic targets in angiogenesis, specifically Serum Kinase Element (SKE) and Aldehyde Dehydrogenase 18 Family Member A1 (ALDH18A1). Venous lung tumor ECs exhibited upregulation of genes related to prostaglandin metabolism, a critical factor in vascular regulation, sprouting, and inflammation. Additionally, nucleotide catabolism-related genes were upregulated, leading to reduced nucleotide content in interferon-activated TECs (Rohlenova et al., 2020). Numerous RNA-seq studies by Vila et al. revealed downregulation of previously identified sprouting-tip EC markers such as Sirpa and Chst1 in epithelial VEGFa mutant lungs compared to controls (Vila et al., 2020). Jambusaria et al. analyzed the dynamics of EC inflammatory responses across tissues, discovering that lung endothelium exhibited significant upregulation of genes associated with immune-related biological processes, including leukocyte cell-cell adhesion, T cell activation, leukocyte migration, and regulation of immune system functions. They identified the ten most significantly upregulated genes in PECs: Sftpc, Advanced Glycosylation End-Product Specific Receptor (Ager), Slc6a2, Chitinase-Like 3 (Chil3), WAP Four-Disulfide Core Domain 2 (Wfdc2), C-Type Lectin Domain Containing 7A (Clec7a), Muc1, Resistin Like Alpha (Retnla), Lysozyme (Lyz), and Homeobox A5 (Hoxa5) — uncovering the most upregulated inflammatory pathways involving chemokines, early immune response mediators, hematopoiesis genes, and cellular stress responses. *In vitro* scRNA-seq revealed that Lymphocyte Antigen 96 (Ly96) was markedly upregulated while Caspase 6 (Casp6) was strongly downregulated in PECs treated with lipopolysaccharide (LPS) for 6 h. Moreover, Forkhead Box F1 (Foxf1) and Tetraspanin 8 (Tspan8) expression levels significantly decreased in PECs treated with LPS for 6 and 24 h before gradually returning to baseline (Jambusaria et al., 2020). These studies underscore the capacity of scRNA-seq to identify up or downregulated genes in PECs, which hold potential as diagnostic and prognostic markers.

### 4.2 Identification of disease-related PECs subclusters

ScRNA-seq facilitates precise cell type definition through transcriptome analysis, enabling the identification of novel subclusters and their corresponding marker genes (Perico et al., 2021). ScRNA-seq data analyses have revealed subclusters associated with lung-related diseases. Additionally, the roles of specific disease-associated EC subclusters have been extensively investigated. Research indicates that PECs undergo phenotypic changes in response to various pulmonary pathologies, including IPF, COPD, and COVID-19 (Li et al., 2021; Terpos et al., 2020; Goshua et al., 2020; Delorey et al., 2021).

Vieira, B.F. et al. identified a novel EC population characterized by high expression of interferon-stimulated genes (ISGs), known for their antiviral properties. These ISG^+^ ECs were situated near infected epithelial cells, suggesting a potential role in curbing viral spread (Vieira et al., 2019). Travaglini et al. discovered a novel EC population with elevated Programmed Death Ligand 1(PD-L1) expression, a ligand for the immune checkpoint receptor Programmed Death 1 (PD-1). PD-L1^+^ ECs were predominantly found in IPF lungs and interacted with PD-1^+^ T cells, indicating their potential to modulate immune responses (Travaglini et al., 2020). Hennigs et al. conducted transcriptomic and immunohistochemical analyses, comparing public RNA-seq and protein datasets. This approach unveiled a specific P2 receptor calcium (Ca^2+^) signalosome in human lung endothelium associated with pulmonary arterial hypertension (Hennigs et al., 2019). In single-cell sequencing analysis of PECs in lung tumors, the study revealed that classical tip and proliferating ECs constituted a minor fraction of the tumor’s EC population. Furthermore, it identified distinct subclusters of tumor ECs linked to basement membrane disruption, immune cell recruitment, and semi-professional antigen presentation (Goveia et al., 2020). ScRNA-seq has revealed that approximately 15% of PECs constitute a transcriptionally distinct subpopulation defined by the expression of Car4. Trajectory analysis further elucidated the developmental divergence of this subset. Functional studies demonstrated that genetic ablation of Car4-expressing ECs, resulting from epithelial-specific deletion of VEGFa, leads to a marked expansion of alveolar spaces, despite the preservation of normal myofibroblast architecture—highlighting the critical role of this endothelial subset in alveolar morphogenesis (Sabbagh et al., 2018).

In the context of lung adenocarcinoma presenting as subsolid pulmonary nodules, scRNA-seq profiling revealed divergent transcriptional programs among endothelial subpopulations. ECs expressing immune-related genes, such as Baculoviral IAP Repeat-Containing 3 (BIRC3), CCL2, CD44, and ICAM1, were enriched in pathways associated with immune activation and lymphocyte recruitment. In contrast, tumor-associated ECs predominantly expressed pro-angiogenic and extracellular matrix remodeling genes, including Heparan Sulfate Proteoglycan 2 (HSPG2) and Periostin (POSTN). Pathway enrichment analysis indicated that Endothelin-1(EDN1)^+^CCL2^+^ endothelial subsets were primarily involved in inflammatory signaling cascades, including TNF-α and interferon-γ (IFN-γ) pathways, whereas tumor-associated endothelial populations exhibited upregulation of biosynthetic and metabolic pathways related to angiogenesis and stromal remodeling (Xing et al., 2021). Additionally, subsets of ECs expressing EDNRB and Interleukin 1 Receptor Like 1 (IL1RL1) demonstrated elevated transcriptional activity of immune-modulatory genes, including ICAM1/2, IL32, and MHC-II molecules, further supporting their role in immune surveillance and activation within the tumor microenvironment. A novel angiogenic pathway centered on collagen modification has also been proposed through the integration of single-cell transcriptomic datasets from multiple human malignancies, corroborated by bulk transcriptome and meta-analytical data as well as functional validation studies. This newly characterized pathway provides mechanistic insight into endothelial remodeling in the tumor vasculature and represents a potential therapeutic target for anti-angiogenic interventions (Goveia et al., 2020). Furthermore, scRNA-seq has identified EC subsets implicated in the pathogenesis of breast cancer, characterized by distinct transcriptional signatures associated with lipid metabolism and immunoregulation. These cells appear to be responsive to metformin, an indirect activator of the PPAR-γ signaling axis, suggesting potential avenues for pharmacological modulation (Geldhof et al., 2022). Collectively, these findings underscore the heterogeneity and functional specialization of PEC subclusters, and highlight their emerging roles in disease pathogenesis, immune regulation, and therapeutic responsiveness. The characterization of these endothelial subsets holds substantial promise for improving diagnostic precision and prognostic assessment in pulmonary and systemic malignancies.

### 4.3 Elucidating the crosstalk between PECs and other pulmonary cell types

**FIGURE 2:**
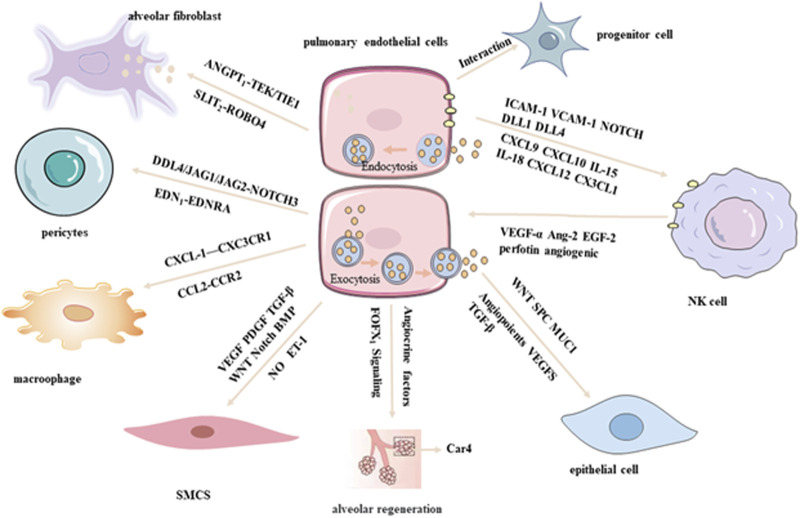
Crosstalk between PECs and other lung cells by scRNA-seq analysis.

PECs engage in dynamic and reciprocal interactions with various resident cell types within the lung microenvironment, including epithelial cells, immune cells, fibroblasts, and smooth muscle cells. These interactions are mediated through direct cell–cell contact as well as paracrine signaling pathways, and play critical roles in regulating lung development, maintaining tissue homeostasis, and contributing to disease pathogenesis (Kumar and Rao, 2020; Marshall et al., 2018). To dissect the molecular underpinnings of these intercellular communications, single-cell regulatory network inference and clustering analyses have been employed, which summaried in Figure 2. These advanced computational approaches enable the identification of distinct cellular phenotypes and the elucidation of signaling crosstalk between PECs and other pulmonary cell populations, offering novel insights into the complex regulatory networks that govern lung physiology and pathology (Zhang et al., 2020).

#### 4.3.1 Crosstalk between PECs and epithelial cells

PECs and epithelial cells are intricately integrated within the alveolar-capillary unit, a critical anatomical site for efficient gas exchange. Recent scRNA-seq studies have revealed substantial heterogeneity and plasticity within both PEC and epithelial cell populations under physiological and pathological conditions. Notably, transcripts typically associated with epithelial identity, such as Spc and Muc1, have also been detected in PECs, indicating a significant degree of molecular interplay between the pulmonary endothelium and parenchymal epithelium (Jambusaria et al., 2020). In a comprehensive analysis conducted by Vieira et al. scRNA-seq profiling of lung cells from both healthy and influenza-infected murine models identified 58 transcriptionally distinct cell populations, comprising 17 endothelial and 14 epithelial subtypes. Coordinated transcriptional alterations were observed between endothelial and epithelial compartments during infection, suggesting dynamic and functionally relevant intercellular communication between these two lineages (Vieira et al., 2019). PECs and epithelial cells share common developmental origins and are regulated by overlapping molecular pathways, including the Wnt signaling axis, which governs their differentiation, spatial organization, and functional integration. Furthermore, their crosstalk is mediated by a repertoire of secreted factors, such as angiopoietins, VEGFs, and TGF-β, which collectively modulate cellular proliferation, survival, and barrier function (Song et al., 2021).

Importantly, PECs serve as a source of angiocrine signals that actively contribute to alveolar regeneration. Endothelial-derived cues have been shown to promote epithelial repair and regeneration, while postnatal alveologenesis is critically dependent on Forkhead Box F1 (FOXF1) signaling within c-KIT^+^ endothelial progenitor cells (Ding et al., 2011; Ren et al., 2019). These findings underscore the essential role of endothelial–epithelial crosstalk in orchestrating lung development, maintaining tissue homeostasis, and facilitating repair following injury.

#### 4.3.2 Crosstalk between PECs and VSMCs, pericytes, and fibroblasts

PECs engage in multifaceted and highly regulated interactions with vascular smooth muscle cells (VSMCs), pericytes, and fibroblasts, playing an indispensable role in maintaining vascular homeostasis, structural integrity, and orchestrating tissue remodeling. Through the secretion of bioactive vasoactive mediators—such as NO (a key vasodilator), and ET-1 (a potent vasoconstrictor)—PECs exert direct regulatory control over the contractile dynamics and phenotypic modulation of VSMCs (Born et al., 2023). In a comprehensive single-cell transcriptomic study, Travaglini et al. profiled lung tissue from three human donors with localized neoplastic lesions, delineating 58 transcriptionally distinct cellular clusters encompassing all major pulmonary lineages, including 10 endothelial and 4 VSMC subtypes. Utilizing the CellPhoneDB algorithm, a robust computational tool for inferring ligand–receptor interactions from high-dimensional gene expression data, the authors identified extensive intercellular communication between ECs and VSMCs mediated via key signaling pathways, including VEGF, PDGF,TGF-β,Wingless/Integrated (WNT), Neurogenic locus notch homolog (NOTCH), and Bone Morphogenetic Protein (BMP).

Notably, pulmonary arterial ECs demonstrated a higher density and complexity of ligand–receptor interactions with VSMCs relative to pulmonary venous ECs, reflecting their discrete developmental origins and functional specialization within the pulmonary vasculature (Travaglini et al., 2020). Building upon these findings, Schupp et al. further elucidated the role of EC subpopulations within the pulmonary cellular interactome, emphasizing critical endotheli–mesenchymal crosstalk. Specifically, arterial ECs were shown to communicate with mural pericytes and VSMCs through the DLL4/JAG1/JAG2–NOTCH3 signaling axis, as well as via the vasoconstrictive EDN1–EDNRA axis. In addition, alveolar fibroblasts were identified as key stromal regulators of endothelial stability, providing essential trophic support to PECs through paracrine signaling pathways including ANGPT1–TEK/TIE1 and SLIT2–ROBO4 (Schupp et al., 2021). Moreover, in the setting of pulmonary fibrosis (PF), scRNA-seq has revealed dysregulated lipid metabolism signatures within specific PEC subsets, suggesting that aberrant endothelial lipid processing may contribute to pathological fibrogenesis. Collectively, these data underscore the complexity and functional relevance of PEC-mediated intercellular communication in the maintenance of vascular architecture and the pathogenesis of pulmonary disease. mmune surveillance and activation.

#### 4.3.3 Crosstalk between PECs and macrophages/progenitor cells

Recent analyses of human control lung tissues using scRNA-seq have revealed intricate interactions between PECs and macrophages, mediated through diverse ligand–receptor pairs such as CX3CL1–CX3CR1 and CCL2–CCR2. These molecular interactions are thought to play a pivotal role in modulating inflammatory responses within the pulmonary microenvironment, thereby contributing to both immune homeostasis and the pathogenesis of lung disease (Schupp et al., 2021). Such findings underscore the power of scRNA-seq in elucidating the complexity of PEC–macrophage communication at single-cell resolution, offering critical insights into the spatial and temporal regulation of immune–endothelial interactions. This growing body of evidence not only enhances our understanding of pulmonary immunobiology but also provides a foundation for the development of targeted therapies aimed at modulating endothelial–immune cell crosstalk in both physiological and pathological contexts. Moreover, the interaction between PECs and progenitor cell populations has garnered increasing attention. Lukowski et al. employed single-cell transcriptomic profiling of the murine aorta to identify two transcriptionally distinct endothelial subpopulations and demonstrated a close association with resident progenitor cell compartments (Lukowski et al., 2019). These observations suggest a fundamental role for PECs in orchestrating vascular repair and regeneration, highlighting their dual function as regulators of both immune responses and vascular progenitor dynamics.

#### 4.3.4 Crosstalk between PECs and NK cells

PECs orchestrate highly specialized and context-dependent interactions with natural killer (NK) cells under a spectrum of physiological and pathological conditions, including viral infections, allergic airway inflammation, pulmonary arterial hypertension, and pulmonary neoplasia. (Zhu et al., 2022; Sauler et al., 2022). PECs play a pivotal immunomodulatory role by regulating NK cell activation, differentiation, and trafficking through both direct intercellular contact and the secretion of soluble mediators. A high-resolution scRNA-seq study conducted by Xing et al. demonstrated that PECs exhibit robust expression of adhesion molecules such as ICAM-1 and vascular cell adhesion molecule-1 (VCAM-1). These molecules interact with integrins on the surface of NK cells, thereby promoting their adhesion to the endothelium and facilitating their transendothelial migration into the pulmonary parenchyma. In addition, PECs secrete chemokines such as CXCL9 and CXCL10, which establish chemotactic gradients that preferentially recruit CXCR3^+^ NK cell subsets. Beyond recruitment, PECs are also instrumental in modulating NK cell activation states. This is achieved through the induction of activation markers, including CD69 and CD25, via both antigen-independent mechanisms—such as the secretion of pro-inflammatory cytokines like antigen-independent interleukin-15 (IL-15) and interleukin-18 (IL-18) and antigen-dependent interactions involving the presentation of MHC class I molecules. Furthermore, scRNA-seq analyses of subsolid nodules in lung adenocarcinoma have elucidated the presence of transcriptionally distinct PEC subpopulations, some of which express chemotactic ligands such as CXCL12 and CX3CL1. These ligands engage corresponding receptors CXCR4 and CX3CR1 on NK cells, further enhancing immune cell recruitment and spatial organization within the tumor microenvironment (Xing et al., 2021; Cheng et al., 2017). Collectively, these findings underscore the multifaceted role of PECs as critical immunoregulatory sentinels within the lung microenvironment and emphasize the power of single-cell transcriptomic technologies in resolving endothelial heterogeneity with high precision. A more refined understanding of PEC–immune cell crosstalk holds significant potential for identifying novel therapeutic targets aimed at modulating immune dynamics in both pulmonary homeostasis and disease pathogenesis.

### 4.4 Reconstructing the developmental and differentiation trajectories of PECs

Trajectory analysis represents a powerful application of scRNA-seq in elucidating the lineage hierarchies and dynamic transcriptional landscapes of PECs. By capturing temporal gene expression changes at single-cell resolution, this approach enables the reconstruction of developmental trajectories, thereby offering critical insights into the ontogeny, functional maturation, and phenotypic plasticity of PECs during embryogenesis, tissue homeostasis, and disease progression (Jia et al., 2021). Trajectory inference facilitates the delineation of endothelial differentiation from progenitor cell populations, the acquisition of organ and vascular bed-specific identities, and the cellular responses to pathophysiological stimuli such as inflammation, injury, and EndMT (Won et al., 2022). Notably, a recent study by Li et al. employed trajectory analysis to characterize PECs in murine embryonic lungs across multiple developmental stages. The study identified two ontogenetically distinct progenitor sources—mesodermal and endodermal lineages—contributing to PEC heterogeneity. Mesodermal progenitors predominantly gave rise to arterial and venous endothelial lineages, whereas endodermal progenitors were mainly responsible for the formation of capillary PECs. Moreover, the study elucidated key transcriptional regulators and signaling pathways governing progenitor fate specification and endothelial subtype differentiation (Li et al., 2022).

Among the most widely utilized computational frameworks for trajectory reconstruction are pseudotime analysis and RNA velocity, which enable high-resolution mapping of cellular transitions and directional lineage progression. These methods facilitate the identification of intermediate cell states, regulatory switches, and lineage bifurcations, thereby advancing our understanding of cell fate decisions and endothelial plasticity (Hwang et al., 2018; Slovin et al., 2021). For instance, Monocle-based trajectory analysis revealed that PECs expressing Plvap at embryonic day 17 (E17) undergo progressive maturation, with a subpopulation transitioning into Car4-expressing PECs along a distinct developmental trajectory. This finding supports a model in which Car4^+^ PECs emerge from Plvap^+^ precursors by embryonic day 19 (E19), highlighting the temporal dynamics of PEC subtype specification (Vila et al., 2020). Collectively, trajectory analysis provides a robust framework for decoding the molecular events underlying PEC differentiation, enabling the identification of critical regulatory genes and transitional states. These insights are instrumental for advancing our understanding of vascular development and remodeling, as well as for identifying potential therapeutic targets in vascular-associated pulmonary diseases (Papalexi and Satija, 2018).

## 5 Discussion

Clarifying the biological characteristics of disease-associated cells is essential for a comprehensive understanding of pathophysiological mechanisms (Van de Sande et al., 2023; Wienke et al., 2024). Integrated scRNA-seq profiling facilitates the identification of biochemical functions in PECs, monitoring of transcription factors and markers, prediction of cell targets for circulating hormones, local signaling interactions, immune cell homing, and direct identification of subclusters impacted by lung disease genes or respiratory viruses. The advancement of scRNA-seq techniques has provided novel insights into the pulmonary microenvironment in both health and disease. For instance, scRNA-seq has been instrumental in evaluating pulmonary microenvironmental heterogeneity and the progression of lung-related diseases. In early-stage lung adenocarcinoma, such as ground-glass nodules, scRNA-seq has detected tumor-associated fibroblasts, immature EC populations, and the emergence of tip-like ECs contributing to the pulmonary tumor microenvironment (Kim et al., 2022).

ScRNA-seq is a powerful tool that has unveiled key molecular mechanisms underlying lung development and pathogenesis (Xia et al., 2023). A deeper understanding of these mechanisms promises to yield predictive models, diagnostic signatures, prognostic biomarkers, and critical information for cancer care, ultimately improving patient outcomes. Furthermore, scRNA-seq will advance prevention strategies and the development of therapeutic agents targeting multiple stages of malignant processes effectively. Current scRNA-seq research is evolving to integrate molecular data not only at the single-cell level but also across diverse cell types and organs with enhanced spatial and temporal precision. Rozenblatt-Rosen et al. developed the Human Tumor Atlas Network (HTAN) based on single-cell resolution, which is poised to significantly contribute to understanding malignancy evolution, metastatic disease, therapeutic responses, and resistance—encompassing both tumor-specific and universal mechanisms (Rozenblatt-Rosen et al., 2020). In the context of precancerous conditions, HTAN is anticipated to facilitate the identification of genetic, epigenetic, and environmental factors associated with early malignancies. This includes distinguishing non-autonomous factors relevant to immune surveillance. In advanced-stage cancers, these atlases may elucidate differences between immune-infiltrated (‘hot’) and immune-deserted (‘cold’) tumor microenvironments, provide insights into metastasis drivers more discernible via spatial data compared to genomic data alone, and examine the influence of tumor heterogeneity and microenvironmental ecosystems on therapeutic response and resistance (Rozenblatt-Rosen et al., 2020).

This approach not only enhances our fundamental understanding of lung biology but also aids in developing innovative diagnostic and therapeutic strategies for pulmonary diseases. Single-cell sequencing technology enables the identification of ECs across different organs and discovery of their specific molecular markers (Du et al., 2024). Furthermore, it facilitates the integration of correlations between ECs across various organs, potentially offering new insights into the organism’s physiopathological state. For instance, the transcriptional profile of ECs is significantly influenced by their physiological functions and tissue environments in adult stages. Organs with similar functionalities or proximal anatomical locations often exhibit correlated gene expression profiles. For example, ECs in the heart and PECs show notable similarities, likely attributable to their adjacent anatomical positions (Feng et al., 2019).

In the future, integrating scRNA-seq with single-cell Assay for Transposase Accessible Chromatin with high-throughput sequencing (scATAC-seq) and other single-cell techniques such as spatial transcriptomics and proteomics will provide a more comprehensive understanding of molecular and cellular mechanisms underlying lung physiology and pathology. This approach will enable exploration of phenotypic changes in PECs during lung-related diseases, identification of transcriptomic and functional disparities among PEC subclusters, and design of drugs targeting specific PEC phenotypes. Such advancements will be instrumental in developing targeted therapeutic agents.
